# Chronic Unpredictable Mild Stress Aggravates Mood Disorder, Cognitive Impairment, and Brain Insulin Resistance in Diabetic Rat

**DOI:** 10.1155/2018/2901863

**Published:** 2018-12-03

**Authors:** Hui Yang, Wei Li, Pan Meng, Zhuo Liu, Jian Liu, Yuhong Wang

**Affiliations:** ^1^Pharmacology Research Center, First Affiliated Hospital, Hunan University of Chinese Medicine, Changsha, Hunan, China; ^2^The Domestic First Class Construction Discipline of Chinese Medicine in Hunan University of Chinese Medicine, Changsha, Hunan, China; ^3^Key Laboratory of Chinese Material Medical Power and Innovation Drugs Established by Provincial and Ministry, Hunan University of Chinese Medicine, Changsha, Hunan, China; ^4^Institute of Innovation and Applied Research, Hunan University of Chinese Medicine, Changsha, Hunan, China

## Abstract

Diabetes-induced brain insulin resistance is associated with many mental diseases, including depression. Epidemiological evidences demonstrate the pathophysiologic link between stress, depression, and diabetes. This study was designed to determine whether chronic unpredictable mild stress- (CUMS-) induced changes in brain insulin resistance could contribute to deterioration in mood and cognitive functions in diabetic rats. Male SD rats were randomly assigned to three groups, including standard control group, the diabetes group, and the diabetes with CUMS group. After 7 weeks, emotional behaviors and memory performances as well as metabolic phenotypes were measured. In addition, we examined the changes in protein expression related to brain insulin signaling. Our results show that rats in diabetes with CUMS group displayed a decreased locomotor behavior in open-field test, an increased immobility time in forced swim test, and tail suspension test, and an impaired learning and memory in the Morris water maze when compared to animals in diabetes group. Further, diabetes with CUMS exhibited a significant decrease in phosphorylation of insulin receptor and an increase phosphorylation of IRS-1 in brain. These results suggest that the depression-like behaviors and cognitive function impairments in diabetic rats with CUMS were related to the changes of brain insulin signaling.

## 1. Introduction

More than 382 million patients worldwide suffered from diabetes mellitus in 2013, and this number will reach 592 million in 2035 [1]. The prevalence of depression in people with diabetes may run up to 38.75%. Among these people, 48.38% were found suffering from moderately depressed [2]. A cross-sectional study [3] described that 35.1% patients with diabetes were diagnosed depression symptoms. Statistically, Bhattacharya and his colleagues found that “total healthcare expenditures were reduced by treatment with antidepressants (16% reduction), psychotherapy (22%), and both therapy types in combination (28%) compared to no depression treatment” [4]. However, the government did not pay attention to the increase prevalence of depression and diabetes and its health services. Moreover, the treatments to patients with related diseases were insufficient [5]. The potential biopsychosocial risk factors of diabetes and depression were the influences on marital status, occupation, and social support [6]. The researches associated with pathophysiological mechanisms of depression in patients with diabetes were insufficient. The association between depression and insulin resistance (IR) can explain the biological link between depression and type 2 diabetes [7]. Understanding the disordered systemic IR and defective brain insulin signaling in comorbidity of diabetes and depression has become a topic of concern and public health challenge.

Comorbidity between diabetes and depression was related to biological, psychological, and social factors, according to Tesfa Dejenie Habtewold [6]. An average depression symptoms score of patients with diabetes was usually affected by external factors including income, educational status, physical activity, and fearing diabetes-related complication and death [6, 8]. However, it is difficult to analyze the external factors in animal trials. Although db/db mice and streptozotocin- (STZ-) diabetic rat were utilized to study this comorbidity [9, 10], the outside influential factors were not considered in most of studies. Chronic unpredictable mild stress (CUMS) procedure has been widely utilized in the study with encouraging results [11]. CUMS model of depression has good predictive validity, face validity, and construct validity [11]. Thus, it is worthwhile to optimize the experimental model in this comorbidity by combining CUMS with diabetes. And it is meaningful to explore the association between depression and diabetes mellitus based on this animal model.

Insulin regulates glucose uptake and storage in peripheral tissues, and it has been shown to alter brain function and metabolism [12–14]. Pancreas is the only organ to produce insulin and the insulin crosses the blood-brain barrier (BBB) by using a saturable transporter, and acts on the brain function through glucose utilization [15].

Previous reports demonstrate that IR leads to memory impairment and it is a risk factor for diabetic encephalopathy in recent years [16, 17]. There are many theories to explain the connection between diabetes and psychiatric disorders, including abnormal glucose metabolism, impaired brain insulin signaling, neurogenesis, and alterations in glucocorticoid levels [18]. Nevertheless, the pathologic role of brain insulin in hippocampus in diabetes-related depression is not fully explained.

A combination of high-fat diet (HFD) and tail meridians injection of streptozotocin (STZ) is a well-established approach to inducing diabetes mellitus [19]. And CUMS provides the diabetic rats with appropriate stress stimulation. However, the possible effect of CUMS on depression-like alterations in rats with diabetes characterized by brain insulin resistance has not yet been investigated. This study is aimed to study the changes of emotional behaviors and memory performances, glucose metabolism, and brain insulin signaling in rats with diabetes subjected to CUMS.

## 2. Material and Methods

### 2.1. Animals

Adult male Sprague-Dawley rats (weight, 200-220g; license No. SCXK 2009-0004) were provided by Hunan Slac Jingda Laboratory Animal Co., Ltd. (Changsha, China). They were housed with access to food and water, and maintained on a 12h light/dark cycle (lights on at 7:00 a.m.), at 22°C with low humidity. All animal experiments were carried out in accordance with the National Institute of Health Guide for the Care and Use of Laboratory Animals (NIH publication 8023, revised 1996) and with the approval of the Animal Ethics Welfare Committee of the First Affiliated Hospital of Hunan University of Chinese Medicine.

### 2.2. Chemicals and Reagents

Streptozotocin (STZ) was purchased from Sigma-Aldrich (St. Louis, MO, USA). The high-fat diet (HFD) consisted of 58% fat, 25% protein, and 17% carbohydrate, as a percentage of the total kilocalories. Antibodies against pIR(Y1158) and pIRS1(S307) were purchased from Abcam (Cambridge, UK).

### 2.3. Instruments

The high-speed refrigerated centrifuge was from Sigma-Aldrich (SIGMA 3K15, Sigma Laborzentrifugen GmbH, Osterode am Harz, Germany), a microplate reader was obtained from Thermo Fisher Scientific Inc. (Waltham, MA, USA; MK3), and open boxes and the Morris water were obtained from Panlab(SMART3.0, Panlab, Spanish).

### 2.4. Model of Diabetes and the Procedure of CUMS

The experimental model of diabetes mellitus (DM) was induced with a combination of low-dose STZ and a HFD. Following the onset of the experiment, the rats were fed libitum with a HFD for two weeks and then received 38 mg/kg STZ freshly dissolved in citrate buffer (pH 4.5) intraperitoneally after fasting overnight [20]. The rats with nonfasting plasma glucose levels of ≥300 mg/dl were considered diabetic and selected for further study. The CUMS model was established according to the methods of Willner with modifications [21]. The stress procedure contained a range of stressors, which consisted of 24 h water deprivation, a 1 min tail pinch, 5 min thermal stimulation in a 45°C oven, 5 min cold swimming at 4°C, a 24 h reversed light/dark cycle, 48 h food deprivation, electric shock to the foot (10 mA current; administered every other minute and lasting 10 sec per time for 30 times), shaking (once per second; lasting for 15 min), noise (85 dB), and strange smell. During a period of 28 days, one of the stimuli was selected randomly and applied to the rats so that the rats were not able to expect the stimulus. Every stimulus used 2 or 3 times in total for each rat within 28 days. So, SD rats were divided into 3 groups, including control group, diabetic group, and diabetes with CUMS group. Body weights were assessed weekly starting from the seventeenth day.

### 2.5. Glucose Metabolism Measurements

#### 2.5.1. Oral Glucose Tolerance Test (OGTT)

Following the final behavioral test, overnight-fasted rat was given a glucose solution orally (2.0 g/kg). And then blood sample was collected from the tail tip of conscious rat before and after glucose load at 0, 30, 60, 90, and 120 minutes for measurements of serum glucose using a single touch glucometer.

#### 2.5.2. Blood Glucose, HbA1C, Insulin, and Leptin

After the oral glucose tolerance test, a single touch glucometer (OneTouch Ultra2; LifeScan, High Wycombe, UK) was used to determine the glucose levels in plasma collected from the tail vein of the rats. Subsequently, the rats were anesthetized, and blood samples were collected by the abdominal aortic method in tubes containing EDTA and centrifuged at 2,500xg for 15min at 4°C. The serum was stored at -70°C until analysis. The serum levels of fasting insulin (FINS, Nanjing Jiancheng, Nanjing, China), glycosylated hemoglobin (HbA1c, Nanjing Jiancheng, Nanjing, China), and leptin (LEP, ELISA LAB, Wuhan, China) were detected using enzymatic kits (Nanjing Jiancheng, Nanjing, China). The homeostasis model assessment of insulin resistance was calculated as followed: (HOMA-IR) = (FPG×FINS) /22.5. All serum samples were measured with a RT-1904C Semi-Auto Chemistry Analyzer (Rayto Life and Analytical Sciences Co., Ltd., Shenzhen, China).

### 2.6. Behavioral Measurements

#### 2.6.1. Open-Field Test (OF)

An 80cm×80cm×40cm open-field was utilized in this experiment. The bottom of the box was divided into 25 equilateral squares. The rats were placed in the central of the open-field, after that the horizontal movement (four feet within a square counted as one score) and vertical movement were counted by scoring within 3min after 1min adaptation.

#### 2.6.2. Tail Suspension Test (TST)

The tail suspension apparatus consisted of an iron shelf supporting a stainless steel bar approximately 30 cm from the ground. About 4 cm tail tip of rats was fixed to that steel bar. Then each rat could adapt to this new condition for 1 minute. Depression-like behavior was inferred from increased duration of immobility in 3 minutes. In addition, all rats could not interfere with each other in the test.

#### 2.6.3. Forced Swim Test (FST)

This test needs a circular fiberglass pool containing warm water (25±1°C). And in this experiment, all the rats were given 1 minute to adjust and 3 minutes to swim. Immobility time was determined by the time a rat stopped struggling. Moreover, moved slowly to remain floating in the water was seen as immobility.

#### 2.6.4. Morris Water Maze Test (MWM)

The Morris water maze consisted of a circular fiberglass pool (200cm in diameter) filled with water (25±1°C) and made opaque with black nontoxic paint. The trials were conducted once a day for five days. The time for rats to locate the platform was recorded. Each trial lasted either until the rat located the platform for 60 sec, which was recorded as the escape latency (EL) time, and the mean EL time of the last four days as the outcome of learning. The platform was removed for a 60-sec probe trial on the final day, and the time spent swimming in the platform quadrant was recorded as the space exploration time (SET).

### 2.7. Western Blot Analysis

The hippocampus was homogenized in lysis buffer (Sigma, St. Louis, MO, USA)]. After that, the protein was electrophoretically resolved on 10% SDS-polyacrylamide gels and transferred to nitrocellulose membranes. The blots were blocked with skimmed milk and incubated in anti-p-IR (1:1000; Cell Signaling Technology, USA) and anti-p-IRS-1 (1:1000; Cell Signaling Technology, USA) for 4°C overnight respectively. And then secondary HRP antibody was added. Finally, the signals were visualized by use of Enhanced Chemioluminescence kit (ECL, Amersham).

### 2.8. Statistical Analysis

All the data were based on SPSS16.0 and analyzed by one-way analysis of variance (ANOVA). Covariance analysis was utilized in evasive latency in water maze test. A level of* p*<0.05 was set as statistically significant.

## 3. Result

### 3.1. CUMS Accelerate Weight Loss in Rat with Diabetes

**Figure 1:**
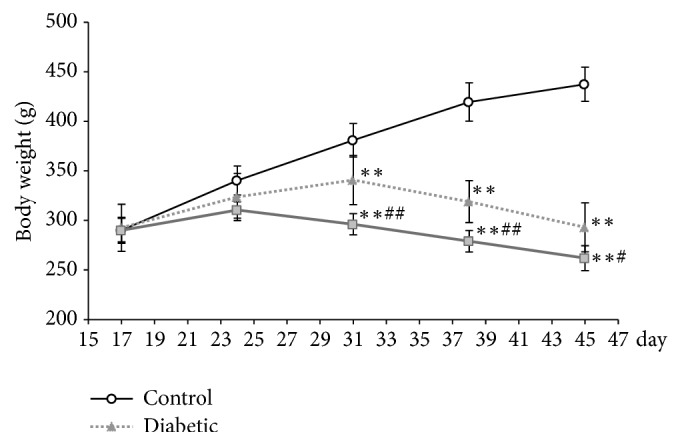
Body weight. Body weight significantly decreased in both diabetic rats. And there was a significant difference between diabetic rat with CUMS and without CUMS. *∗∗p*<0.01, control versus diabetic. #*p*<0.05, diabetic versus diabetic +CUMS. ##*p*<0.01, diabetic versus diabetic +CUMS.

Rats in normal group kept a healthy weight gain during the whole experiment, while diabetic rats put on a little weight at first 2 weeks and lost weight at last 2 weeks. However, when CUMS was started, the weight loss was the most serious. Starting from 31 days, significant weight loss was observed in both diabetic groups compared with the control group (Figure 1,* p*<0.01). Meanwhile, a serious weight loss was found in diabetes with CUMS rather than diabetic rats (Figure 1,* p*<0.05 and* p*<0.01).

### 3.2. Disorders of Blood Glucose and Relative Indexes in Diabetic Rats with CUMS

**Figure 2:**
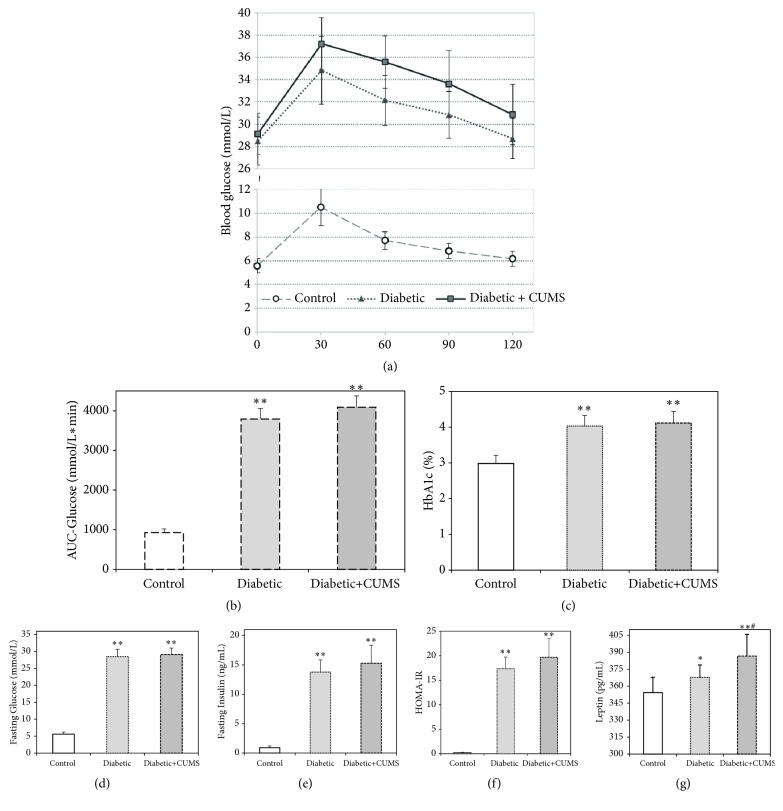
Characterization of diabetic rats with CUMS. High-fat diet and streptozotocin injection induces changes in fasting blood glucose levels, HbA1c levels, insulin resistance index, and oral glucose tolerance test. After receiving CUMS, there were no obvious changes in the above indexes of the diabetic rats. (a) Oral glucose tolerance test. (b) Area under curve of the glucose level in oral glucose tolerance test. (c) The level of HbA1c. (d) Fasting blood glucose concentration. (e) Fasting insulin concentration. (f) Insulin resistance index. (g) The level of leptin. *∗∗p*<0.01, control versus diabetic.

High-fat diet and streptozotocin injection resulted in a diabetic syndrome verified by the presence of hyperglycemia, high levels of hemoglobin A1c concentrations (HbA1c), and peripheral insulin resistance. The concentrations of fasting blood glucose, HbA1c, insulin, and leptin in both of the diabetic groups were significantly higher than the control group (Figures 2(c), 2(d), 2(e), and 2(g),* p*<0.01 or* p*<0.05). Moreover, glucose tolerance and insulin resistance were impaired in diabetic groups than control group (Figures 2(b) and 2(f),* p*<0.01). However, there were no significant differences of blood glucose and relative indexes between diabetic group and diabetic + CUMS group except for leptin.

### 3.3. CUMS Decrease the Performance Status in Diabetic Rat

**Figure 3:**
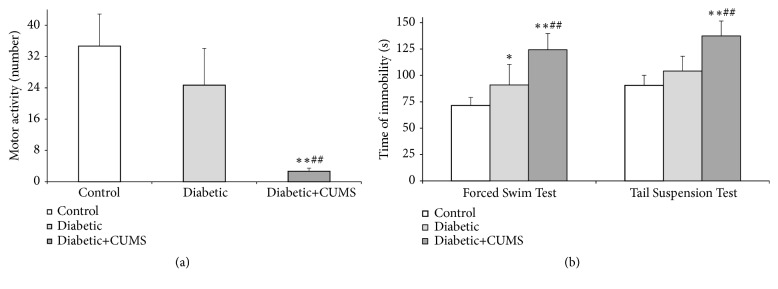
CUMS induces changes in depressive-like behaviors of diabetic rats. (a) The locomotion in OF. (b) The time of immobility in TST and FST. *∗∗p*<0.01, control versus diabetic. ##*p*<0.01, diabetic versus diabetic +CUMS.

Depression-like behaviors were assessed in the open-field (OF), forced swim test (FST), and tail suspension test (TST). In the OF, the horizontal activity and vertical activity were observed to evaluate the motion activity and curiosity in an open-field. The result indicates that there was a down-regulation in both diabetic groups when compared with control group. However, the total activity scores of horizon activities and vertical activities were significantly reduced in diabetic + CUMS group instead of diabetic group when compared with control group (Figure 3(a),* p*<0.01). Further, the diabetic rats with CUMS had fewer activity scores when compared with the diabetic group (Figure 3(a),* p*<0.01). Moreover, in FST, the duration of immobility was obviously increased in diabetic group and diabetic + CUMS group when contrast to control group (Figure 3(b),* p*<0.01 and* p*<0.05). And there was a dramatic difference between diabetes group and diabetes + CUMS group (Figure 3(b),* p*<0.01). Similarly, in TST, the time of immobility was obvious longer in diabetic + CUMS group than it was in control group, and it was much longer than diabetes group as well (Figure 3(b),* p*<0.01). Nevertheless, in this test, there were no significant differences between diabetic group and control group. Thus, the results demonstrate that depression-like behaviors in diabetic + CUMS group were more obvious than other groups.

### 3.4. CUMS Leads to a Declined Capability of Learning and Memory in Diabetic Rat

**Figure 4:**
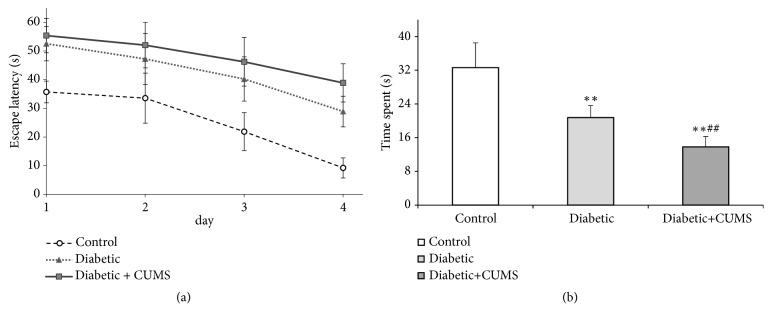
CUMS induces declines in cognitive function of diabetic rats. (a) The time of escape latency. (b) The time of space exploration. *∗∗p*<0.01, control versus diabetic. ##*p*<0.01, #*p*<0.05, diabetic versus diabetic +CUMS.

The purpose of Morris water maze is to test the capability of learning and memory by place navigation and space exploration. The evasive latency (EL) was recorded in place navigation. There was a negative relationship between EL and the duration of the training days in all three groups according to regression analysis (Figure 4(a)). It appears to be a linear relationship (R^2^ value 0.9307, 0.9702, and 0.9742) in control group, diabetic group, and diabetic + CUMS group, respectively. EL went down significantly over time when the animals had high learning capacity. Thus, we use the slope of the regression curve to demonstrate the capability of learning. And we found that there is a significant difference between diabetic + CUMS group and control group in learning slope curve according to covariance analysis (*p*=0.046).

On the 5th day of the test, place exploration was performed. The duration for rats to spend in target area and locate the site (platform) was recorded as space exploration time (SET). The result demonstrates that SET in target area was significantly lower in both diabetic groups when compared with control group (Figure 4(b),* p*<0.01). These data suggest that the capability of learning and memory in diabetic rat were significantly affected by CUMS.

### 3.5. The Abnormal Brain Insulin Signaling Pathway in the Hippocampus of Diabetic Rats with CUMS

**Figure 5:**
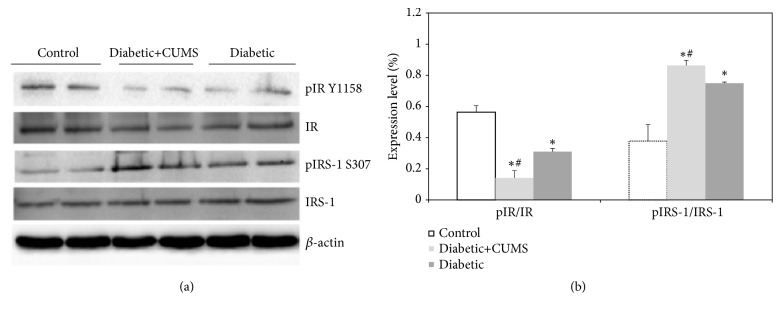
CUMS led to impairment of insulin signaling pathway in hippocampus of diabetic rats. (a) Representative Western blots of IR and IRS-1 proteins. (b) Densitometry measurements. *∗p*<0.05, control versus diabetic. #*p*<0.05, diabetic versus diabetic +CUMS.

The levels of the phosphorylation of IR and Ser phosphorylation of IRS-1 protein were analyzed by a quantitative Western blot procedure in hippocampus (Figure 5(a)). The intensities of *β*-actin bands were taken as an equal load controls and the ratios p-IR: IR and p-IRS-1: IRS-1 were calculated for each lane and the results are expressed as a percentage of p-IR and p-IRS-1 proteins (Figure 5(b),* p*<0.05). It has been found that both of diabetic rats had lower hippocampal p-IR and higher p-IRS-1 concentration when compared with control group. Diabetic rats subjected to CUMS increased in p-IR and decreased in p-IRS-1 levels in hippocampus compared with diabetic rats.

## 4. Discussion

Diabetes usually causes a number of complications involving brain function which related to cognitive decline and depression [22]. The effects of diabetes on central nervous system (CNS) were related to the negative impact of behavioral and emotional functions, with pathological mechanism [9, 10, 23]. The behavioral despair performed an increased immobility time in forced swim test in adult db/db mice [23]. However, Dinel and his colleagues reported that an impaired spatial recognition memory was found in db/db mice, rather than depressive-like behaviors [10]. Moreover, Can et al. found that diabetes mellitus (DM) causes depression deterioration, and spontaneous locomotor activities were decreased accompanied with learning parameters impairment [9]. Thus, the results of mood disturbances (depression) in diabetic rats are inconsistent, as well as it was in this study (Figure 3). Clinical evidences suggest that lots of external factors, such as inactivity, poor sleep, diet, and early life stress are associated with both diabetes and depression [24]. Therefore, we can surmise that the above competing results in animal experiments were related to the reason that external factors were not considered. CUMS is a classic method for building an animal model with a core symptom of depression [11]. This approach can provide a chronic mild stress and simulate the stress that patients suffered from. Thus, in this study, the influence of CUMS on the changes of emotional behaviors and memory performances in diabetic rats was investigated, as well as central insulin signaling.

Prior to the above problems, it is better for us to understand that whether CUMS could affect the body weight, glucose level, and systemic insulin resistance in diabetic rats. There was a statistically significant weight loss in both of diabetic groups (Figure 1). And the increased glucose, HbA1c level, and leptin concentration were found in both of diabetic rat (Figures 2(b), 2(c), and 2(g)), which exhibited impaired glucose tolerance (Figures 2(a) and 2(c)). These results indicate systemic IR with the high HOMA-IR index in two diabetic groups (Figure 2(d)). Leptin is an adipocyte hormone regarded as the afferent signal in a negative feedback loop regulating insulin biosynthesis and secretion [25]. The increased results of the insulin and leptin suggest that the leptin resistant occurred in increased leptin and it caused hyperinsulinemia. And stress could deteriorate the leptin resistant of diabetic rat. Furthermore, there are no significant differences between diabetic and diabetic + CUMS group on the aspect of fasting blood glucose, glucose tolerance, HbA1c, and peripheral insulin resistance (Figure 2). Although previous study shows that an oral glucose tolerance and serum insulin levels in normal control animals were damaged after CUMS was performed [26], we found that there are no remarkable changes in metabolic phenotypes after CUMS performed on diabetic rats in this study. Thus, it is concluded that the effect of outside interfere becomes inconspicuous after the occurrence of diseases such as obesity diabetes with which already accompany disordered endocrine function.

It has been previously studied that diabetes mellitus (DM) have negative impacts on the central nervous system [27–30]. Many literatures suggested that the cognitive impairment was closely related to diabetes [31, 32]. We also observed that there are significant changes in learning and memory performance in diabetic rats with CUMS when compared to control group (Figure 4). However, whether depression-like behaviors are associated with diabetes mellitus is not clear. Liu and his coworkers found that db/db mice performed increased anxiety-like behaviors instead of depression-like behaviors [10]. On the contrary, another research group suggests that diabetes mellitus exacerbate the depression levels [9]. Thus, this study reported the depression behaviors and locomotor deficits in diabetic rats and CUMS with diabetes. Our results reveal that diabetes rats exhibited depressive-like behaviors as assessed by immobility time in the forced swim rather than depression in the open-field and tail suspension tests (Figure 3). Based on this study, it suggests that depressive moods and cognitive deficits do not occur at the same time in diabetes. S. Sasaki-Hamada and his colleagues found that synaptic plasticity of hippocampus was affected by the length of diabetes [33]. In addition, neuroplasticity is thought to be closely related to mood disorders [34]. Consistence to the above results, we found that there is a link between the depression-like behaviors and the length of diabetes in diabetic rats. Furthermore, cognitive impairment, especially memory damage, may occur earlier than mood disorder in diabetic rats. More importantly, CUMS could aggravate the emotional and cognitive impairment in diabetic rats, whereas, as stated before, the imbalanced glucose metabolism is hardly deteriorated after CUMS performed in diabetic rats. It concluded that the effect of interfere stress on behavior and cognition is greater than that of blood glucose on behavior and cognition when diabetes was existed. Thus, ignoring the psychological counseling of diabetic patients may accelerate the occurrence of diabetes-related depression.

Insulin signaling in brain plays an important role in the development and progression of diabetes mellitus [35], as well as diabetic encephalopathy [16]. This system of the brain is involved in the regulation of neuronal growth and synaptic plasticity and controls metabolic process in the CNS and periphery [36]. Depression symptoms and cognitive functions including spatial memory are associated with brain insulin resistance in type 2 diabetes [37, 38]. Furthermore, the recent researches indicate that chronic stress mediated behavioral dysfunction in normal mice are associated with impaired hippocampal insulin signaling [39]. To further investigate the underlying molecular in diabetes, depression, and stress, CUMS performed on diabetes was utilized to induce IR in peripheral and central organs. Our study researched the activation of insulin signaling in the hippocampus, a key brain area for the control of emotional and cognitive behaviors. Insulin receptor and its major downstream targets, insulin receptor substrate 1 (IRS-1), and IRS-2 are regarded as the core in insulin signaling [40]. The different phosphorylated subtypes of IRS family of protein could activate the different downstream signaling cascade, thereby inducing the physiological function and pathological change. For example, phosphorylation of IRS could activate phosphatidylinositol 3-kinase (PI3K) and phosphoinositide-dependent protein kinase-1 (PDK1) activation, thereby activating the downstream signaling cascade involving Akt [41]. Activation of Akt leads to the phosphorylation of GSK3*β*, and the Akt/GSK3*β* pathways are important regulators of depression [42]. In addition, phosphorylated IRS can also regulate the activation of JNK, CHOP (stress), and NF-*κ*B (inflammatory pathways) [43]. Our data demonstrated that phosphorylation of IR was decreased, while serine phosphorylation of IRS-1 was increased in hippocampus in diabetic rats (Figure 5). When CUMS is applied to diabetic animals, the increased p-IPS-1 level and decreased p-IR level were getting severer.

In summary, it is difficult to explain the relationship between diabetes and depression. Recent reports demonstrate that shared clinical and pathophysiologic traits between diabetes and depression raise the possibility that stress and pressure play an important role in the pathophysiology of cognitive decline. Data in this study reveal the effects of CUMS aggravated mood disorder, cognitive impairment in diabetic rats. These results are in accordance with the previous studies that people whom lived in a bad situation would be more prone to depression. Moreover, animals with diabetes are more prone to pose negative effects on brain insulin signaling under CUMS condition.

## Data Availability

All the data used to support the findings of this study are included within the article.
